# Sortilin promotes glioblastoma invasion and mesenchymal transition through GSK-3β/β-catenin/twist pathway

**DOI:** 10.1038/s41419-019-1449-9

**Published:** 2019-02-27

**Authors:** Wei Yang, Peng-fei Wu, Jian-xing Ma, Mao-jun Liao, Xu-hui Wang, Lun-shan Xu, Min-hui Xu, Liang Yi

**Affiliations:** Department of Neurosurgery, Daping Hospital and Institute Research of Surgery, Army Medical University, Chongqing, 400042 China

## Abstract

High aggressiveness is a hallmark of glioblastoma and predicts poor prognosis of patients with glioblastoma. The expression level of sortilin has been preliminarily reported to be elevated in high-grade glioma; however, the potential significance of sortilin in glioblastoma progression has not been elucidated. In this study, we investigated the oncogenic effect of sortilin in glioblastoma. Increased levels of sortilin were noted in the mesenchymal subtype of glioblastoma and highly aggressive subtypes of glioblastoma tissues and cell lines. In addition, high levels of sortilin predicted poor prognoses in patients with glioblastoma. Sortilin knockdown or inhibition with AF38469 (an orally bioavailable inhibitor of sortilin) significantly suppressed migration and invasion by inhibiting EMT-like mesenchymal transition in glioblastoma cells. Furthermore, we proved that sortilin promoted cell invasion mainly via Glycogen synthase kinase 3 beta (GSK-3β)/β-catenin/Twist-induced EMT-like mesenchymal transition in glioblastoma. Taken together, our results demonstrate a critical role of sortilin in glioblastoma invasion and EMT-like mesenchymal transition, indicating that sortilin contributes to glioblastoma progression. These data also highlight the dramatic antitumor effects of AF38469 in glioblastoma, suggesting that AF38469 is a potentially powerful antitumor agent for sortilin-overexpressing human glioblastoma.

## Introduction

Human glioblastoma (GBM) is the most common and aggressive form of malignant primary tumor in the central nervous system (CNS)^1^. Although current multimodal therapeutic strategies for human GBM (including surgical resection, concurrent chemoradiotherapy, and adjuvant temozolomide therapy) have improved patient survival, the prognosis of patients with GBM is still dismal^2–4^. High aggressiveness is a hallmark of human GBM, which makes it difficult to be completely eradicated, resulting in relapse and death in patients with GBM. Although tumor invasion is a hot topic in the field, the mechanisms underlying GBM invasion are still not entirely understood. Therefore, elucidation of the molecular mechanisms underlying GBM invasion and development of novel and effective strategies for GBM treatment are urgently needed.

Epithelial–mesenchymal transition (EMT) has been reported to induce epithelial cells to undergo numerous biochemical changes to switch to a mesenchymal phenotype, defined by an enhanced invasive capacity^5^. Importantly, a recent report has confirmed that the mesenchymal subtype is closely related to the high invasive capacity of GBM^6^. In addition, WNT/β-catenin contributes to mesenchymal transition; WNT and β-catenin are expressed at high levels and are correlated with a significantly short survival time in patients with GBM^7,8^. In general, WNT/β-catenin is activated in GBM and contributes to tumor invasion by triggering the expression of EMT activators such as Twist, Snail, and ZEB1^9^. Furthermore, accumulating evidence indicates that Twist, a downstream activator of WNT/β-catenin, is highly expressed in GBM and promotes cell invasion by regulating the expression of mesenchymal target genes^10,11^.

Our previous work has demonstrated that the overexpression of neurotensin (NTS) is closely linked with human glioma progression. The biological effects of NTS are triggered by its interaction with three distinct receptors NTSR1, NTSR2, and sortilin^12^. Sortilin is a member of the Vps10p sorting receptor family, which has important roles in various biological processes, such as transporting intracellular proteins, acting as a co-receptor for the 75 kDa neurotrophin receptor (p75^NTR^) or receptor tyrosine kinases (RTKs), and acting as a regulator of atherosclerosis^13,14^. Elevated expression of sortilin has been found in high-grade glioma and is positively correlated with the malignancy of glioma, suggesting that sortilin might have an important role in the progression of human glioma^15^. However, the potential significance of sortilin in GBM has not been elucidated.

In this study, we investigated the expression levels of sortilin in the mesenchymal, classical, proneural, and neural subtypes of GBM. Bioinformatics analysis predicted that the expression level of sortilin was elevated in the mesenchymal subtype and a negative correlation was found between sortilin levels and the prognosis of patients with GBM. We used AF38469 (a novel, selective, and orally bioavailable inhibitor of sortilin) to block the effects of sortilin on cell motility and mesenchymal transition in GBM^16^. We found that AF38469 inhibited GBM invasion mainly through Glycogen synthase kinase 3 beta (GSK-3β)/β-catenin/Twist-induced mesenchymal transition in vitro and in vivo. Our results suggest that sortilin contributes to GBM progression and maybe a novel prognostic factor for GBM. Foremost, AF38469 could be developed as a promising therapeutic agent for human GBM.

## Materials and methods

### Human tissue specimens and cell culture

Five human GBM tissues (including intratumor (IT) region, peritumor (PT) region, and normal brain (NB) region) were obtained from patients who are undergoing surgery at the Department of Neurosurgery, Daping Hospital of Army Medical University. Informed consent was obtained from all patients and the study was approved by the Ethics Committee of the Daping Hospital of Army Medical University. These GBM tissues were graded by at least two experienced clinical pathologists according to the World Health Organization classification system. GBM cell lines Rat (C6), Human (SHG44, U87, A172, LN229, and U251), Mouse (GL261), and Human embryonic kidney cell line (HEK293T) were purchased from American Type Culture Collection. Short tandem repeat (STR) confirmation was conducted by the Institute Research of Surgery of Army Medical University. All cell lines were cultured in Dulbecco’s modified Eagle’s medium/F12 containing 10% fetal bovine serum and incubated at 37 ℃ in a humidified incubator with 5% CO_2_.

### Reagents and antibodies

AF38469 was purchased from MedChem Express. SB216763, dimethyl sulfoxide (DMSO), and 4′,6-diamidino-2-phenylindole (DAPI) were obtained from Sigma Aldrich. Lipofectamine 2000 was obtained from Invitrogen. Fluorescein isothiocyanate (FITC)-phalloidin was purchased from Life Technological. Rabbit anti-sortilin monoclonal antibody was purchased from GeneTex. Anti-GSK-3β, p-GSK-3β, β-catenin, T-cadherin, N-cadherin, vimentin, MMP-2, MMP-9, Twist, and Actin antibodies were purchased from CST.

### Analysis of patient data

Kaplan–Meier analysis was conducted with the Gravedeel dataset and the Tumor GBM-TCGA-540 dataset from R2: Genomics Analysis and Visualization Platform (RGAVP), which includes 273 glioma cases and 485 cases of de novo GBM, respectively. The expression level of sortilin was investigated with the Gravedeel dataset from RGAVP, which includes 273 glioma cases with different histological grades. The expression levels of sortilin in four subtypes of human GBM were analyzed with the Tumor GBM-TCGA-540 dataset. The resulting BoxPlot, survival curves, and *p*-values were downloaded from the RGAVP.

### Lentivirus vector construction, infection, and siRNA transfection

We purchased LV-Ctr and LV-Twist from Genechem, The GBM cells were infected with lentivirus particles or control lentivirus particles. Puromycin was used to induce stable cell line for subsequent assays. Small interfering RNA (siRNA) targeting sortilin, GSK-3β, and twist were purchased from RibiBio (RibiBio, Co., China). The GBM cells were transfected with siRNA and cultured for 48 h before performing the assays according to the manufacturer’s instructions.

### Cell migration and invasion assays

Cells were seeded in six-well plates at 90% confluence and starved for 12 h. A wounding line was scratched with 10 μL pipet tip and the serum-free medium was added to the plates. Cells were incubated for 24 h. The migrated cells were monitored with Olympus inverted microscope and counted in five randomly selected fields to quantify the cell migration area. Transwell filters (8.0 μM pore size, Corning) were pre-coated with Matrigel (Corning). The cells were starved and 5 × 10^4^ cells in 100 μL of serum-free medium were seeded into the upper chamber. The lower chamber was filled with 600 μL of medium containing 5% fetal bovine serum. After 24 h of incubation, cells were removed from the upper surface. Invaded cells on the lower surface were fixed, permeated, and stained with 5% crystal violet (Beyotime). Invaded cells were counted in five randomly selected fields to quantify the invasive rate.

### Western blotting assay

Cells were washed in phosphate-buffered saline and lysed in ice-cold lysis buffer for the whole-cell protein. The equal amounts of proteins were separated by SDS-polyacrylamide gel electrophoresis and transferred onto polyvinylidene difluoride membrane. The membrane was blocked and incubated with primary antibodies. Specific antibodies against sortilin (1:3000), against GSK-3β, p-GSK-3β, β-catenin, T-cadherin, N-cadherin, vimentin, and Actin (1:1000), against MMP-2 and MMP-9 (1:5000), and against Twist (1:3000) were used to detect the corresponding protein. The membrane was washed and incubated with secondary antibodies. Immunoblots were detected by Western-Bright Sirius HRP Substrate Kit (Advansta) and exposed to ChemiDoc TMXRS + Image System (Bio-Rad). The Image J was used to analyze the relative expression level of protein. To detect the change of β-catenin expression in different subcellular compartments, we extracted membranal protein, cytoplasmic protein, and nuclear protein from whole-cell lysate by Nuclear and Cytoplasm Protein Extraction Kit (Beyotime) and Mem-PERTMPlus Membrane Protein Extraction Kit (ThermoFisher), then preformed western blotting assays to detect β-catenin and analyzed the results by Image J software. Antibody of β-catenin, PCNA, Na^+^-K^+^-ATPase, and Actin were diluted to 1:1000. We calculated the three independent results by SPSS 13.0 and built graph by GraphPad 5.0.

### Quantitative real-time PCR

Total RNA extracted from the cultured cells by TRIzol reagent (Invitrogen) cDNA was reverse transcribed from 1.0 μg RNA with Prime Script RT Reagent Kit (TAKALA). The mRNA levels of sortilin, GSK-3β, β-catenin, twist, and glyceraldehyde 3-phosphate dehydrogenase (GAPDH) detected by Real-time PCR was performed with SYBR Premix Ex TaqTM Kit (TAKALA). The result was detected at CFX 96 Real-Time PCR Detection System (Bio-Rad). Fold changes in relative gene expression were calculated by comparative Ct method (fold change = 2^−ΔΔCt^). Primers for PCR are as follow: SORT1:F:5′-GAAGTCGTGGAGGAAGAATCTTT-3′, R:5′-TGGTGTTGTCTGATCCCCATT-3′; GSK-3β:F:5′-GGCAGCATGAAAGTTAGCAGA-3′, R:5′-GGCGACCAGTTCTCCTGAATC-3′; β-catenin:F:5′-AAAGCGGCTGTTAGTCACTGG-3′, R:5′-CGAGTCATTGCATACTGTCCAT-3′; Twist:F:5′-GTCCGCAGTCTTACGAGGAG-3′, R:5′-GCTTGAGGGTCTGAATCTTGCT-3′; GAPDH:F:5′-ATTGACCTCAACTACATGGTTTACATG-3′, R:5′-TTGGAGGGATCTCGCTCCTGGAAG-3′.

### Immunohistochemistry and immunofluorescence

Human GBM tissues were fixed and cut into 5 μm sections. The slides were treated for antigen retrieval and were incubated with primary and secondary antibodies, and enzyme conjugate horseradish peroxidase. Cells on cover slips were fixed, blocked, and permeated, then incubated with primary antibodies (sortilin 1:100; GSK-3β 1:200; β-catenin 1:200) and fluorescence-labeled secondary antibodies. The nucleus was stained with DAPI and examined with Zeiss LSM 780 Meta confocal microscope. The images were processed by ZEN software.

### Subcutaneous and orthotopic xenografts implantation and MRI scan

Immunodeficient athymic nude mice were purchased from the Experimental Animal Center of Army Medical University. Ectopic subcutaneous GBM transplantation model in nude mice was established by injection of 1 × 10^6^ U87 cells. The mice in the AF38469 group (*n* = 6) were intraperitoneally injected with AF38469 diluted in DMSO (0.01 g/day/kg) and the mice in control group (*n* = 6) were injected with same value of DMSO. The orthotopic xenografts model in nude mice was established by injection of 1 × 10^7^ U87 cells into the right striatum of mice brains (*n* = 12). Magnetic resonance imaging (MRI) (Broker Biospec 7.0 Tesla Imaging System) was performed to detect tumor dimensions. After the first MRI detection to confirm successful transplantation, 12 mice was divided into two group, then intraperitoneally injected with AF38469 and same value of DMSO. All animal experiments were performed in accordance with the Guidelines for the Care and Use of Laboratory Animals, and all experimental protocols were approved by the Institutional Animal Care and Use Committee of Army Medical University.

### Statistical analysis

Statistical analyses were carried out using SPSS 13.0 statistical software. Statistical significance was calculated by Student’s *t*-test or one-way analysis of variance. Survival was analyzed by Kaplan–Meier method and compared by log-rank test by GraphPad Prism 5; *p < 0.05 was considered statistically significant.

## Results

### Sortilin expression is elevated in highly invasive GBM subtypes and negatively correlated with patient prognosis

**Fig. 1:**
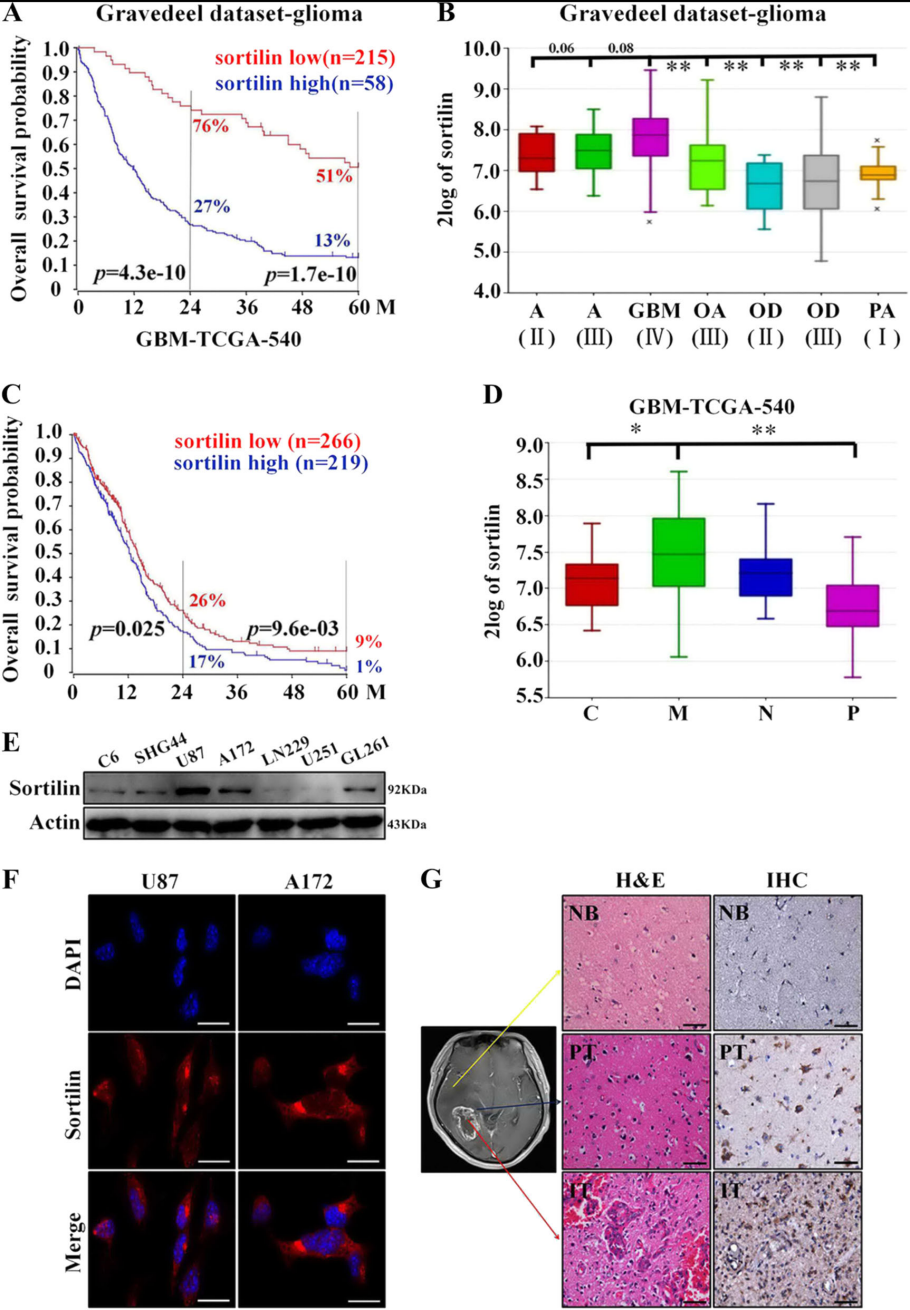
The expression of sortilin is elevated in highly invasive glioblastoma subtypes and negatively correlated with patient prognosis. **a** Kaplan–Meier analysis of the 2-year survival rates and the 5-year survival rates of patients with low level and high level of sortilin from the RGAVP, which includes 273 different grades of glioma cases, *p*-value was determined using the log-rank test. **b** The expression levels of sortilin in different pathological grades of glioma from the RGAVP (A astrocytoma, GBM glioblastoma multiforme, OA oligoastrocytoma, OD oligodendroglioma, PA pilocytic astrocytoma; II, III, IV WHO grade classification of glioma), *p*-value was determined using the independent samples *T*-test (**p < 0.01). **c** Kaplan–Meier analysis of the 2-year survival rates and the 5-year survival rates of patients with low level and high level of sortilin from the Tumor GBM-TCGA-540 dataset (*n* = 485); *p*-value was determined using the log-rank test. **d** The expression levels of sortilin in different subtypes of glioblastoma (C classical subtypes, M mesenchymal subtype, N neural subtype, P proneural subtype) were analyzed on the R2 microarray analysis and visualization platform, *p*-value was determined using the independent samples *T*-test (*p < 0.05, **p < 0.01). **e** Western blotting analysis of sortilin protein expression in glioblastoma cell lines (C6, SHG44, U87, A172, LN229, U251, GL261). **f** Representative immunofluorescence images of sortilin (red) and nucleus (blue) in U87 and A172 cell lines, scale bar = 50 μm. **g** Representative H&E (scale bar = 100 μm), IHC (sortilin, scale bar = 100 μm), and IHC (sortilin combined with Ki67, scale bar = 20 μm) stained slices from the different regions of human glioblastoma specimens (NB normal brain, PT peritumor, IT intratumor).*p < 0.05, **p < 0.01

To preliminarily investigate the relationship between the expression levels of sortilin and the prognosis of glioma patients, we employed the Gravedeel dataset from RGAVP, which includes 273 glioma cases with different histological grades. The microarray date could be downloaded from the Gene Expression Omnibus public database at NCBI (GSE16011). The result confirmed that high Soritlin expression indicated a significantly poor prognosis. In detail, Kaplan–Meier analysis showed that the 2-year survival rates for patients with high expression (58 cases) and low expression (215 cases) of Sortilin mRNA were 27% and 76%, respectively, and the 5-year survival rates for these patients were 13% and 51%, respectively (Fig. 1a). Meanwhile, GBM expressed the highest level of sortilin in comparison with other histological types of glioma. However, the increase of sortilin expression is positively correlated with high-grade astrocytoma (vs. oligodendroglioma and grade I astrocytoma), but there is no significant difference between astrocytoma grades II, III, and IV (Fig. 1b). In addition, we employed TCGA data from RGAVP with the Tumor GBM-TCGA-540 dataset (*n* = 485), which includes 485 cases of de novo GBM. The Kaplan–Meier analysis showed that the 2-year survival rates of sortilin-low (*n* = 266) and sortilin-high (*n* = 219) GBM patients were 26% and 17%, respectively, and the 5-year survival rates were 9% and 1%, respectively (Fig. 1c). All grades of glioma patients were included in Gravedeel dataset and only GBM patients were included in GBM-TCGA dataset. Due to the prognosis of GBM being significantly worse than low-grade glioma, the magnitude of difference in Gravedeel dataset was greater than the GBM-TCGA dataset. Second, to further detect the expression pattern of sortilin in four subtypes of GBM^18^, we employed TCGA data. Here, a significantly higher expression level of sortilin was found in the mesenchymal subtype than in the classical and proneural subtypes; however, there was no significant difference between the mesenchymal subtype and neural subtype (Fig. 1d).

Next, we performed western blotting assays to detect the levels of sortilin in GBM cell lines and observed a high level of sortilin in U87 and A172 cells, which are highly aggressive (Fig. 1e). Furthermore, we investigated the subcellular location of sortilin in U87 and A172 cell lines by immunofluorescence staining, and found that majority of sortilin was located in the cytoplasm and a small part of sortilin was distributed on the membrane (Fig. 1f). Finally, we employed immunohistochemistry (IHC) to detect the levels of sortilin in different regions of human GBM specimens. The results of IHC indicated that the levels of sortilin were obviously higher in the IT region that was characterized by high aggressiveness than in the PT region and NB region NB (Fig. 1g). Taken together, these data suggest that the expression level of sortilin is strongly correlated with the aggressive capacity of GBM, and that high levels of sortilin predict poor prognoses in patients with GBM. Thus, sortilin maybe effectively targeted by an inhibitor to repress GBM progression.

### Sortilin promotes GBM migration and invasion in vitro

**Fig. 2:**
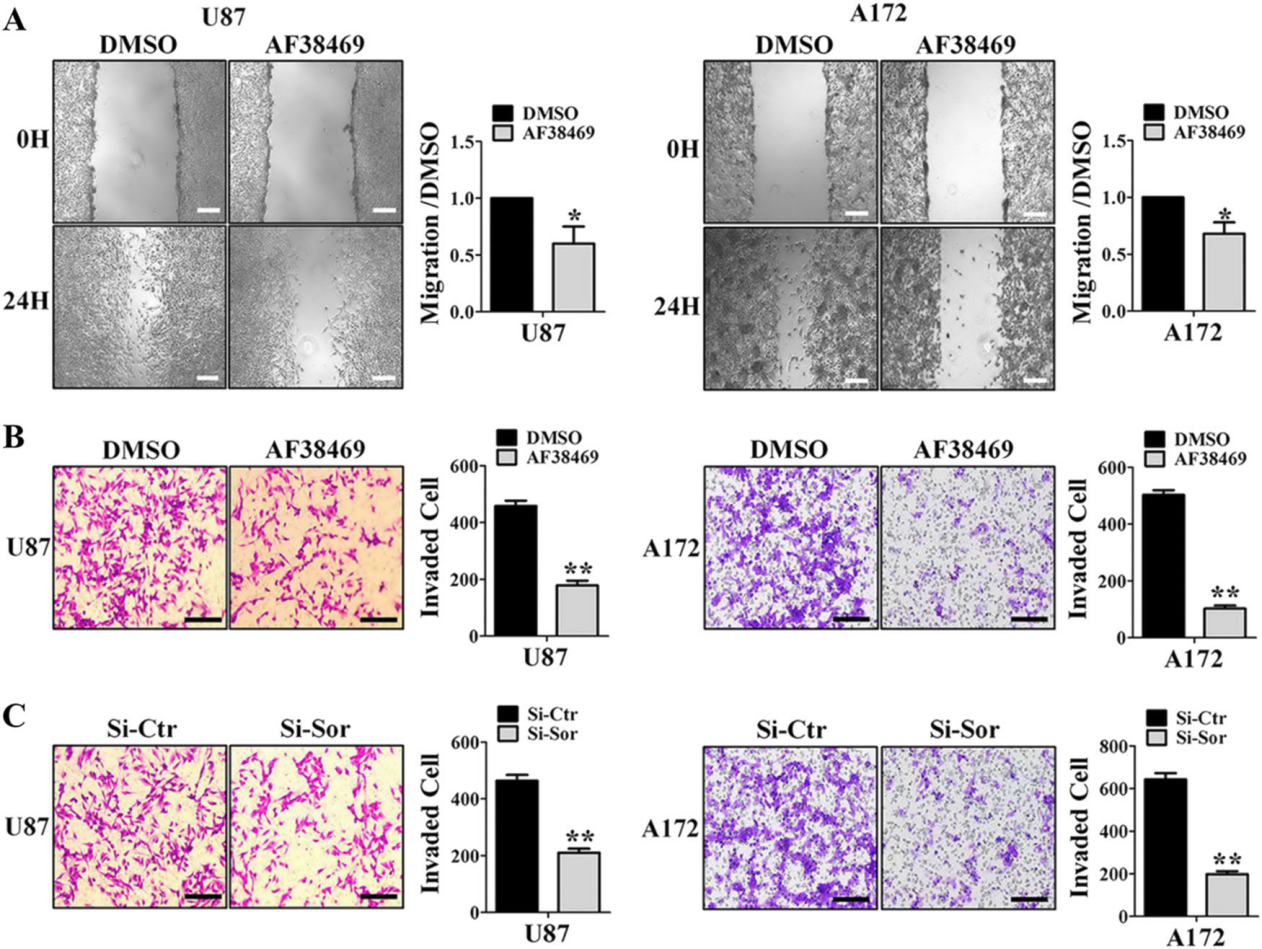
AF38469 represses glioblastoma cells migration and invasion in vitro. **a** U87 and A172 cell lines were treated with 400 nM sortilin inhibitor (AF38469). Cells were scraped and imaged immediately (0 H) and later (24 H); the images of wound gap were taken for analysis; scale bar = 200 μm. **b** U87 and A172 cell lines were treated with 400 nM AF38469. Invaded cells were stained and counted using microscopy; scale bar = 200 μm. **c** U87 and A172 cell lines were transfected with Si-Sor. Invaded cells were stained and counted using microscopy. The histogram corresponds to the mean ± SD of three independent experiment; *p < 0.05, **p < 0.01, scale bar = 200 μm

The above data preliminarily indicate that sortilin is associated with the high aggressiveness of GBM. To investigate the biological effects of AF38469 on GBM cell motility, we performed wound-healing assays. Compared with DMSO, 400 nM AF38469 impaired the migratory capabilities of U87 and A172 cell lines (Fig. 2a). Next, we examined the effect of AF38469 on the invasive ability of GBM cells by transwell assays. As shown in Fig. 2b, AF38469 significantly attenuated the invasive capacities of U87 and A172 cell lines. To provide further evidence of AF38469 effect on GBM, we knocked down sortilin with siRNA targeting sortilin (Si-Sor) (Supplementary Fig. S1A). Consistent with the above results, knockdown of sortilin significantly reduced the invasive abilities of U87 and A172 cell lines (Fig. 2c). We employed transwell assays in U251 cell line, which had low expression of sortilin, and AF38469 and Si-Sor did not influence the invasion ability of U251 cell, which had low expression of sortilin (Supplementary Fig. 1B). These results indicate that sortilin promotes migration and invasion in GBM cell lines, and that AF38469 impairs the invasive ability of GBM by blocking sortilin.

### Sortilin induces an EMT-like mesenchymal phenotype in GBM

**Fig. 3:**
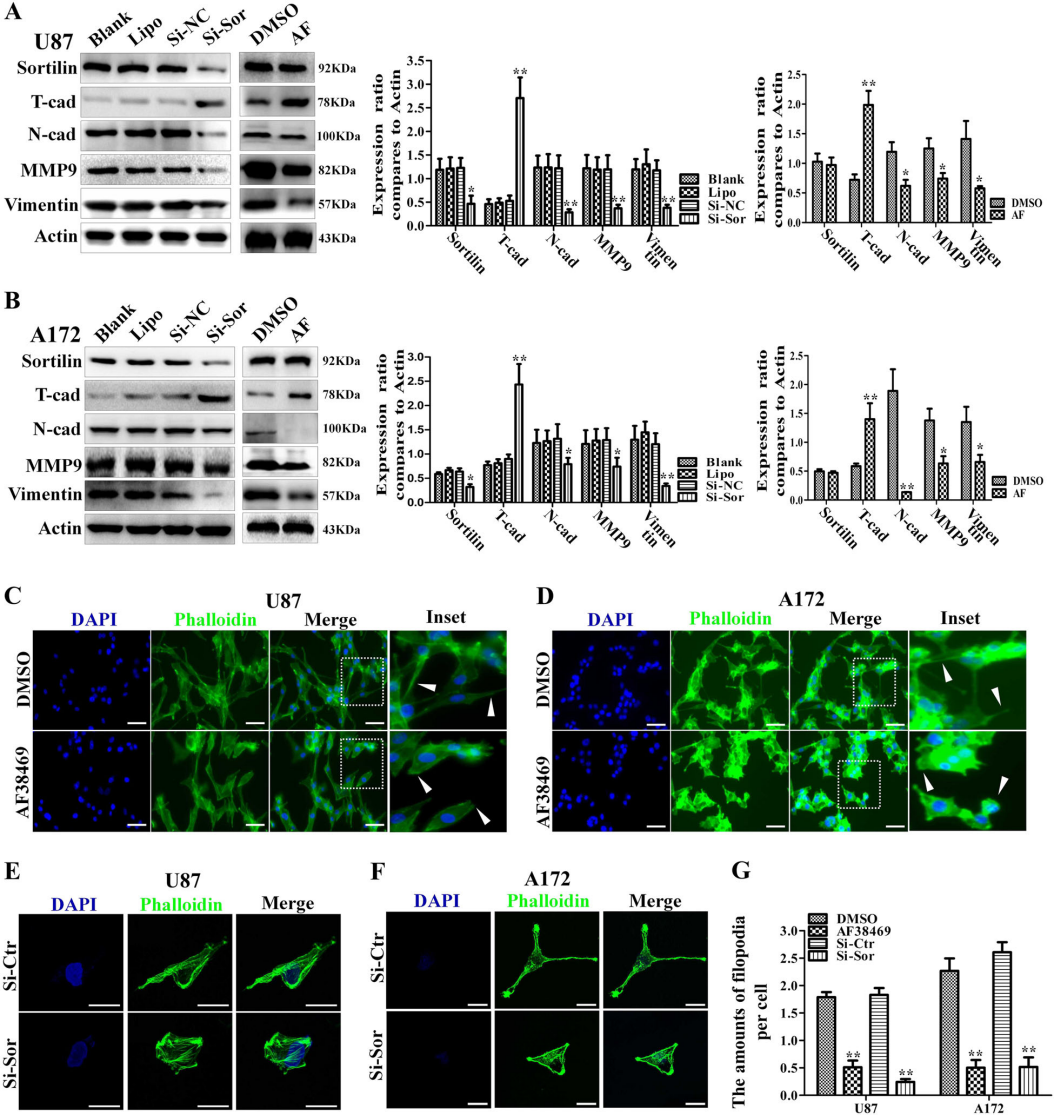
Sortilin promotes the EMT-like mesenchymal transition of glioblastoma. **a**, **b** Western blotting analysis of the expression levels of classical mesenchymal markers (N-cadherin, MMP-9, and vimentin) and T-cadherin, U87, and A172 cell lines that were treated with 400 nM AF38469 or Si-Sor. **c**, **d** Representative immunofluorescence images of phalloidin (green) and nucleus (blue) in U87 and A172 cell lines that were treated with 400 nM AF38469; scale bar = 50 μm. **e**, **f** Representative cellular morphology change of U87 and A172 cell when transfected with Si-Sor. Scale bar = 10 μm. **g** The numbers of filopodia were calculated from five random image of each group and shown in histogram. Error bars (SD) represent the data of triplicate samples for each group. Paired two-way Student’s *t*-test; *p < 0.05, **p < 0.01

EMT-like mesenchymal phenotype is closely associated with the aggressive capacity of GBM and our data indicate that sortilin promotes the invasive ability of GBM. Thus, we speculated that sortilin is involved in the EMT-like transition of GBM. To confirm our hypothesis, we first detected the levels of EMT-related markers such as N-cadherin, vimentin, T-cadherin, and MMP-9 in the U87 cell line. Based on the results of western blotting, the levels of N-cadherin, vimentin, and MMP-9 in the AF38469 groups were markedly lower than those in the control groups; nevertheless, T-cadherin, a glial marker in the CNS, was strongly upregulated after treating with AF38469 (Fig. 3a). We also forced the evidence of above results by Si-Sor knockdown. Furthermore, a similar effect was observed in the A172 cell line (Fig. 3b). These data suggest that sortilin promotes the EMT-like mesenchymal transition of GBM and AF38469 could repress this process by targeting sortilin.

To further investigate the effects of sortilin on EMT-like mesenchymal transition, we investigated the morphology of U87 and A172 cell lines using cytoskeletal markers (FITC-phalloidin). U87 and A172 cells treated with AF38469 were more round and displayed decreased filopodia formation, whereas cells treated with DMSO had spindle-shaped morphology, displayed increase filopodia formation, and were more disorganized than the AF38469-treated cells (Fig. 3c, d). Based on the above changes, the EMT-like transition of GBM cells was considered to be reversed by AF38469^19^. To further confirm these results, we also observed U87 and A172 cells changed their elongated and stretched, fibroblastic morphology toward minimal astrocytic-like morphology after sortilin was blocked by si-Sortilin. The amounts of filopodia sharply decreased after blocking sortilin by AF38469 or si-sor (Fig. 3e–g). Therefore, these data suggest that sortilin is critical for the EMT-like mesenchymal transition of GBM, and that AF38469 reverses the mesenchymal transition of GBM.

### Sortilin positively regulates the GSK-3β/β-catenin/Twist pathway in GBM

**Fig. 4:**
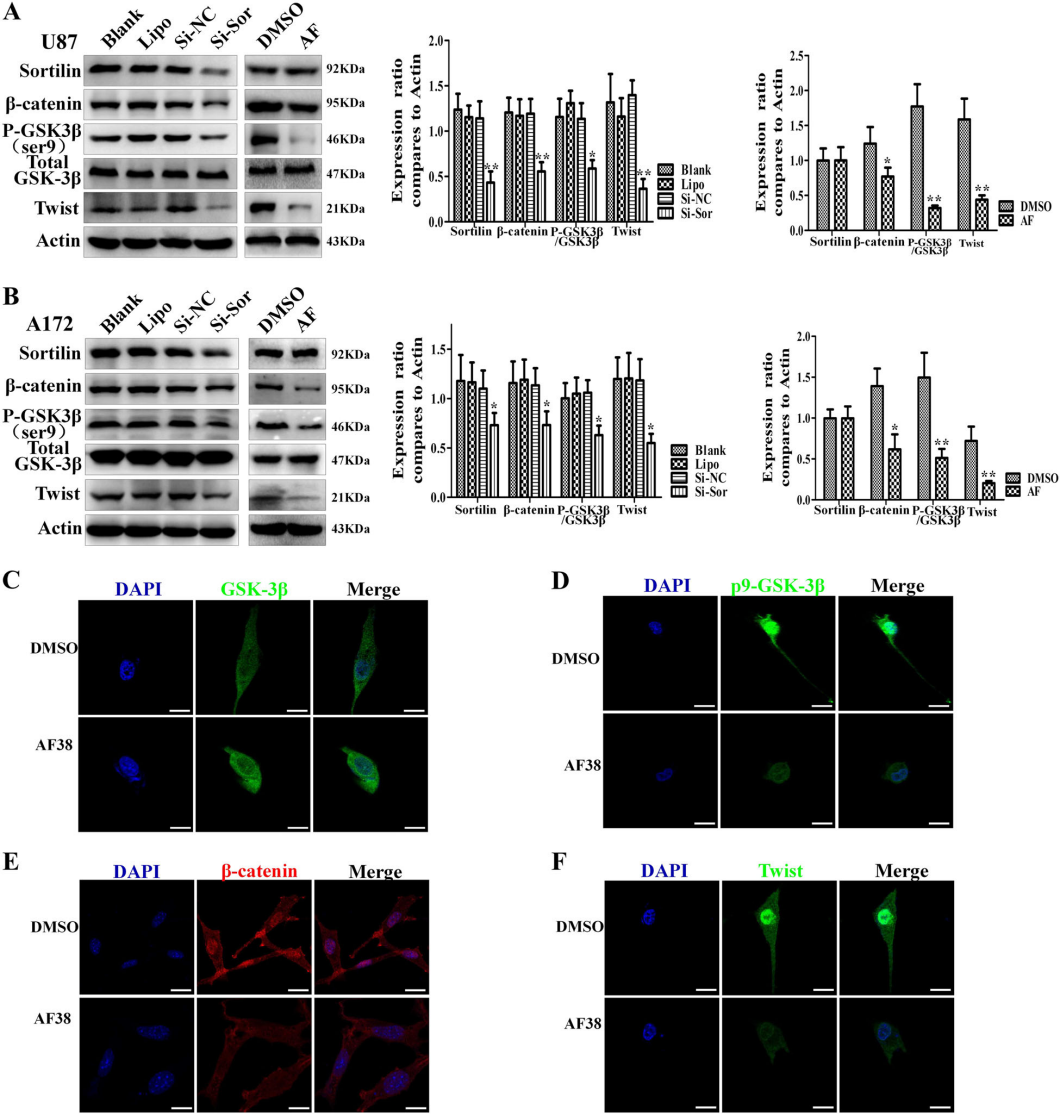
Sortilin positively regulates GSK-3β/β-catenin/Twist pathway in glioblastoma. **a**, **b** Western blotting analysis of the expression levels of sortilin, p-GSK-3β, GSK-3β, β-catenin, and Twist in U87 and A172 cell lines treated with 400 nM AF38469 or transfected with Si-Sor. **c**–**f** Representative immunofluorescence images to show the expression change of GSK-3β, p-GSK-3β, β-catenin, and Twist in U87 cells; scale bar = 10 μm. Error bars (SD) represent the data of triplicate samples for each marker, paired two-way Student’s *t*-test; *p < 0.05, **p < 0.01

**Fig. 5:**
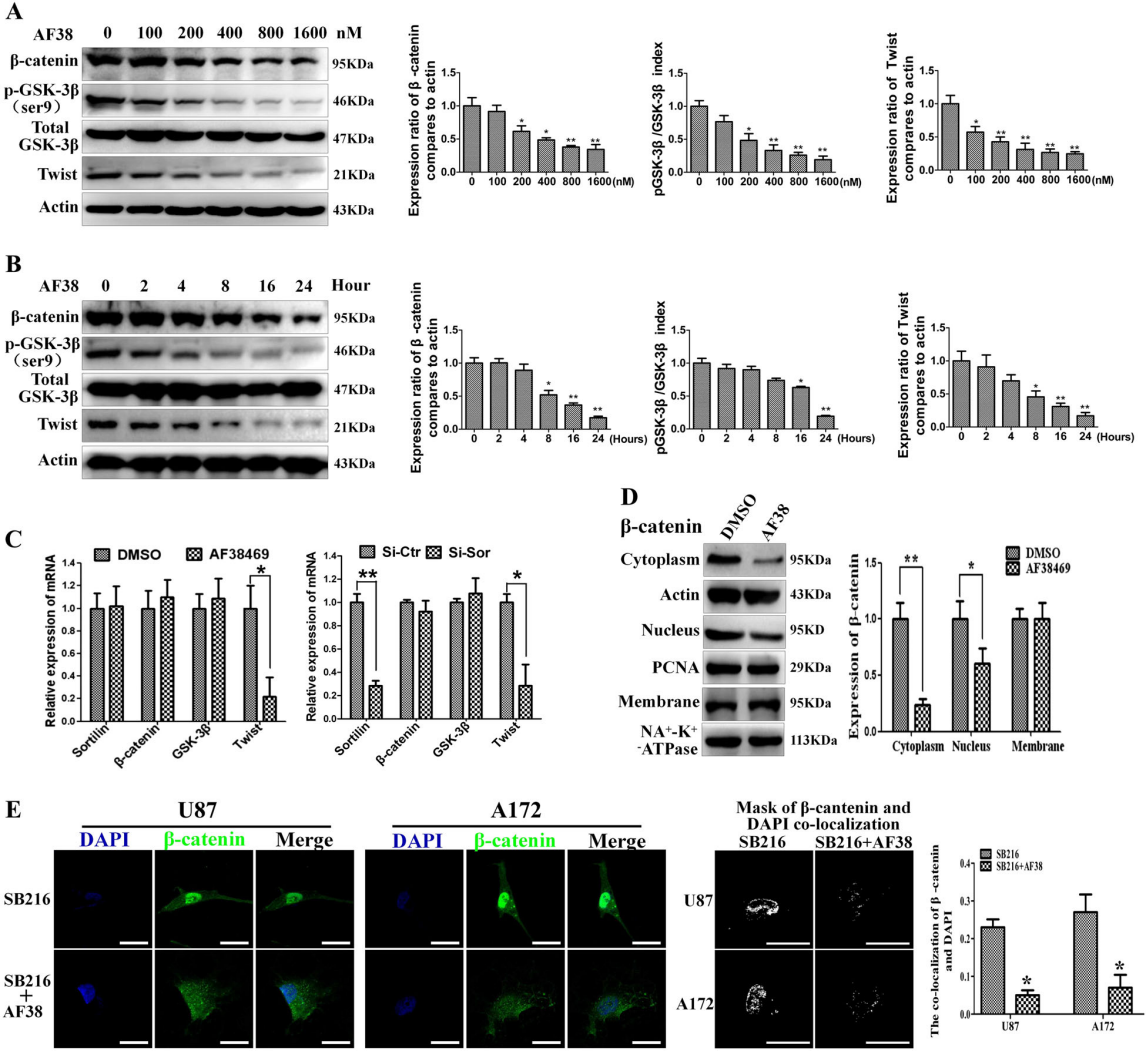
**AF38469 inhibits GSK-3β/β-catenin/Twist by dose- and time-dependent manner and hinders translocation of β-catenin in glioblastoma cells.** **a**, **b** Western blotting analysis of the expression levels of GSK-3β, p-GSK-3β, β-catenin, and Twist with different dose and different time of AF38469 treated in U87 cell. **c** The chenge of mRNA levels of sortilin, GSK-3β, β-cantein, and twist in U87 cells when treated with 400 nM AF38469 or transfected with Si-Sor. **d** Western blotting to investigate the expression change of β-catenin at the cell membrane, cytoplasm, and nucleus in U87 cells treated with 400 nM AF38469. **e** Immunofluorescence images to show the distribution of β-catenin in U87 cells when treated with SB216763 alone or combined with AF38469; the masks representing colocalization of green (β-catenin) to blue (DAPI) were built by Image-pro-plus. The colocalization coefficient were analyzed and displayed. Scale bar = 10 μm. Error bars (SD) represent the data of triplicate samples for each group. Paired two-way Student’s *t*-test, *p < 0.05, **p < 0.01

WNT/β-catenin and Twist have been reported to be critical for EMT-like transition in GBM. To investigate whether sortilin enhances the EMT-like mesenchymal transition of GBM via WNT/β-catenin and Twist, we performed western blotting assays and found that AF38469 decreased the levels of p-GSK-3β, β-catenin, and Twist in U87 and A172 cells. We found similar results when Si-Sor blocked sortilin in both U87 and A172 cells (Fig. 4a, b). Furthermore, we found that the levels of p-GSK-3β, β-catenin, and Twist were decreased in U87 cells with AF38469 treated by immunofluorescence staining, but the expression of total GSK-3β was not significantly decreased with AF38469 treatment (Fig. 4c–f). These data suggest that sortilin positively regulates GSK-3β/β-catenin and Twist in GBM. Furthermore, we found AF38469 regulated GSK-3β, β-catenin, and Twist by dose- and time-dependent manner (Fig. 5a, b), and results from quantitative real-time PCR (QT-PCR) showed that the mRNA levels of twist was sharply decreased by blocking sortilin. However, the mRNA levels of GSK-3β and β-catenin did not obviously change when sortilin was repressed. These results suggested sortlin regulated twist at transcriptional steps, but regulated GSK-3β/β-catenin at posttranscriptional level (Fig. 5c). Furthermore, we found sortilin repression only decreased the cytoplasm and nucleus part of β-catenin, of which the majority participated in WNT signal transduction, but ignored the membrane part of β-catenin, which constructed the cell–cell conjunction with E-cadherin to prevent cell migration (Fig. 5d). Interestingly, we employed GSK-3β inhibitor (SB216763) to relieve the degradation of β-catenin and combined sortilin inhibitor treatment not only decreased the expression of β-catenin but also retarded translocation of β-catenin to the nucleus and detained at the cytoplasm (Fig. 5e). Next, we used SB216763 to promote phosphorylation of GSK-3β, which could not influence the expression of sortilin (Fig. S1C). In addition, we overexpressed Twist by lentivirus and it did not change the expression levels of sortilin, GSK-3β, p-GSK-3β, and twist as well (Fig. S1D). The above data implied that sortilin unidirectionally and positively regulated GSK-3β/β-catenin and Twist axis in GBM cells.

### GSK-3β/β-catenin/Twist-mediated sortilin promoted invasion and EMT-like mesenchymal transition

**Fig. 6:**
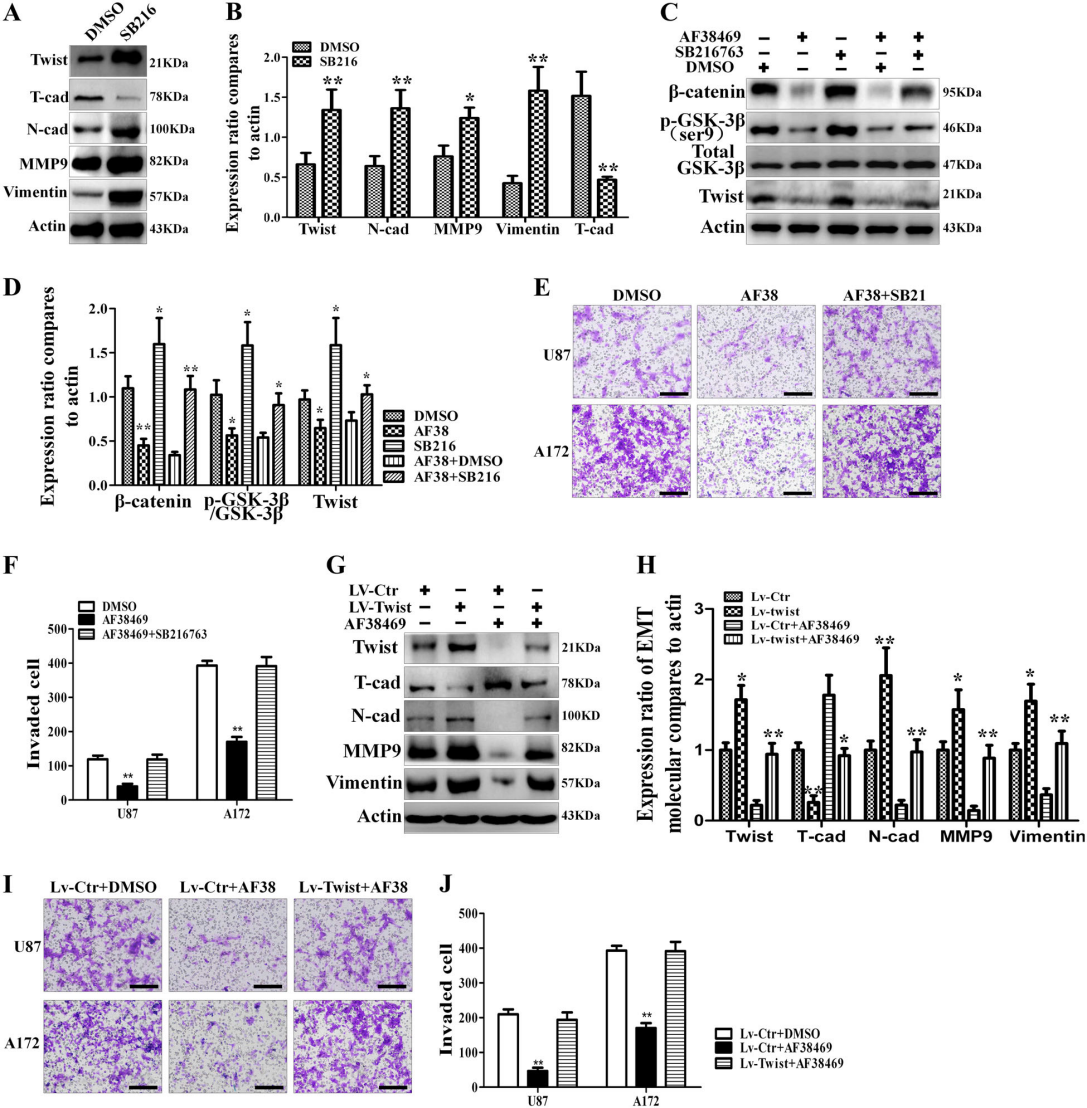
GSK-3β/β-catenin/Twist is critical for sortilin-stimulated invasion and mesenchymal transition. **a**, **b** Western blotting analysis of the expression levels of EMT markers (twist, N-cadherin, MMP-9, vimentin, and T-cadherin) in U87 cells treated with SB216763. **c**, **d** Western blotting analysis of the activation of GSK-3β/β-catenin/twist in the indicated groups of U87 cells. **e**, **f** U87 and 172 cells were treated with AF38469 alone or combined with SB216763. Invaded cells were stained and counted using microscopy. Scale bar = 200 μm. **g**, **h** Western blotting to detect the expression levels of EMT markers (twist, N-cadherin, MMP-9, vimentin, and T-cadherin) in U87 cells after overexpressed twist. **i**, **j** Transwell assays to detect the invasion ability of U87 cells and 172 cells when treated with AF38469 alone or combined with overexpression of twist. Scale bar = 200 μm. Error bars (SD) represent the data of triplicate samples for each group. Paired two-way Student’s *t*-test, *p < 0.05, **p < 0.01

WNT/β-catenin and Twist have been shown to have a vital role in GBM invasion and EMT^18^. Our data confirm that AF38469 negatively modulates GSK-3β/β-catenin/Twist in GBM. To investigate whether GSK-3β/β-catenin/Twist is required for AF38469-inhibited GBM invasion and EMT-like transition, we first treated U87 cells with SB216763 and found this inhibitor significantly decreased T-cadherin expression, while increasing the levels of N-cadherin, vimentin, MMP-9, and Twist, indicating that SB216763 accelerated the EMT-like transition of GBM (Fig. 6a, b). Foremost, SB216763 treatment could alleviate the depression of GSK-3β/β-catenin/Twist axis, which was caused by sortilin antagonist (Fig. 6c, d). Next, we investigated the role of SB216763 in U87 and A172 cell lines. Based on transwell invasion assays, GSK-3β inhibited by SB216763 completely rescued the repression of invasive ability by AF38469 in GBM cells (Fig. 6e, f). After down-expressed GSK-3β by siRNA, the repression of invasion ability, caused by AF38469 treatment, was significantly eliminated in U87 and A172 cells (Fig. S2A). Lastly, we employed U251 GBM cell as a negative control model, which expressed low level of sortilin. Although SB216763 could significantly promote cell invasion of U251 cells, following with AF38469 treatment had no effect on SB216763-promoted invasiveness (Fig. S2C). These results implied that GSK-3β/β-catenin is the downstream signal of sortilin in regulating invasion and mesenchymal transition in GBM. To further identify the correlation between twist and sortilin/GSK-3β/β-catenin, we overexpressed twist in U87 cells by using lentivirus. We found a significant increase of N-cadherin, vimentin, and MMP-9 expression, whereas T-cadherin expression in U87 cells treated by Lv-TWIST decreased. In addition, Lv-Twist completely repressed the AF38469-mediated upregulation of T-cadherin and increased the AF38469-mediated downregulation of N-cadherin, vimentin, and MMP-9 (Fig. 6g, h). According to transwell assay results, upregulated twist entirely counteracted the AF38469-induced repression of cell invasion ability of U87 and A172 cells (Fig. 6i, j). On the contrary, knockdown twist with siRNA completely diminished the depression of invasion ability by AF38469 treatment in U87 and A172 cells (Fig. S2B). In U251 GBM cells, AF38469 treatment followed by overexpression of twist did not change invasiveness (Fig.S2D). Taken together, GSK-3β/β-catenin/Twist transducted the stimulation signal of sortilin to invasion and mesenchymal transition in GBM.

### Targeting sortilin with AF38469 effectively inhibits GBM invasion and EMT-like mesenchymal transition in vivo

**Fig. 7:**
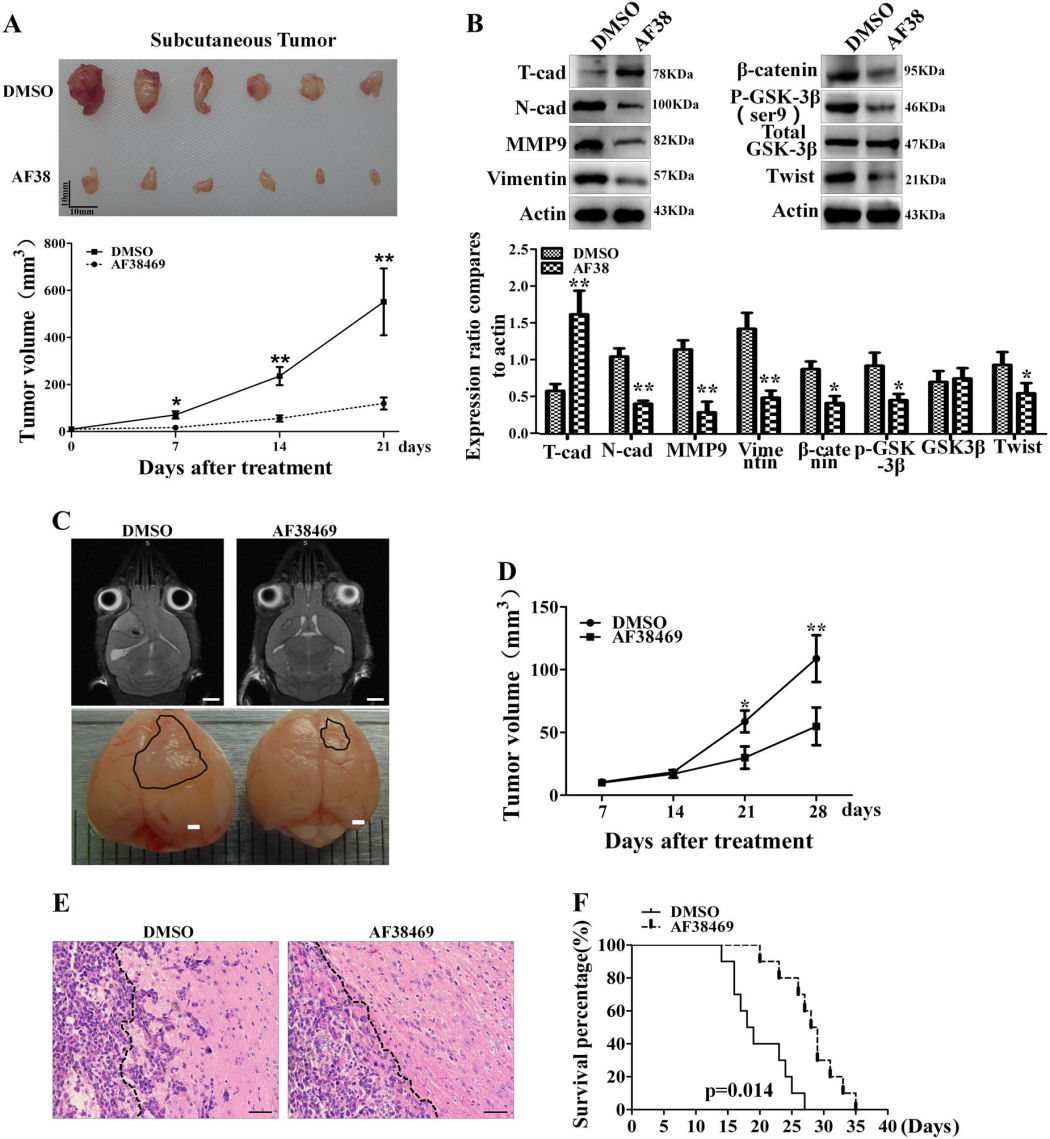
AF38469 inhibits glioblastoma invasion and mesenchymal transition in vivo. **a** The gross observation of subcutaneous glioblastoma at day 21 post injection (Upper) and the volume of tumor were measured and calculated every week after transplanting. Scale bar = 10 mm (*p < 0.05, **p < 0.01). **b** Western blotting analysis of the activation of GSK-3β/β-catenin/twist and the expression levels of EMT markers (N-cadherin, MMP-9, vimentin, and T-cadherin) in subcutaneous glioblastoma tissues. Error bars (SD), paired two-way Student’s *t*-test, *p < 0.05, **p < 0.01. **c** Representative MRI images (scale bar = 2 mm) and gross observations (scale bar = 1 mm) of orthotopic glioblastoma xenografts in the indicated groups. **d** The volumes of orthotopic xenografts were detected by MRI and calculated every week after transplanting. **e** H&E staining to show the margin of orthotopic xenografts, dotted black line to show the border of glioblastoma. Scale bar = 50 μm. **f** The survival time of glioblastoma-bearing mice was analyzed by Kaplan–Meier method and compared by log-rank test. *p < 0.05, **p < 0.01

**Fig. 8:**
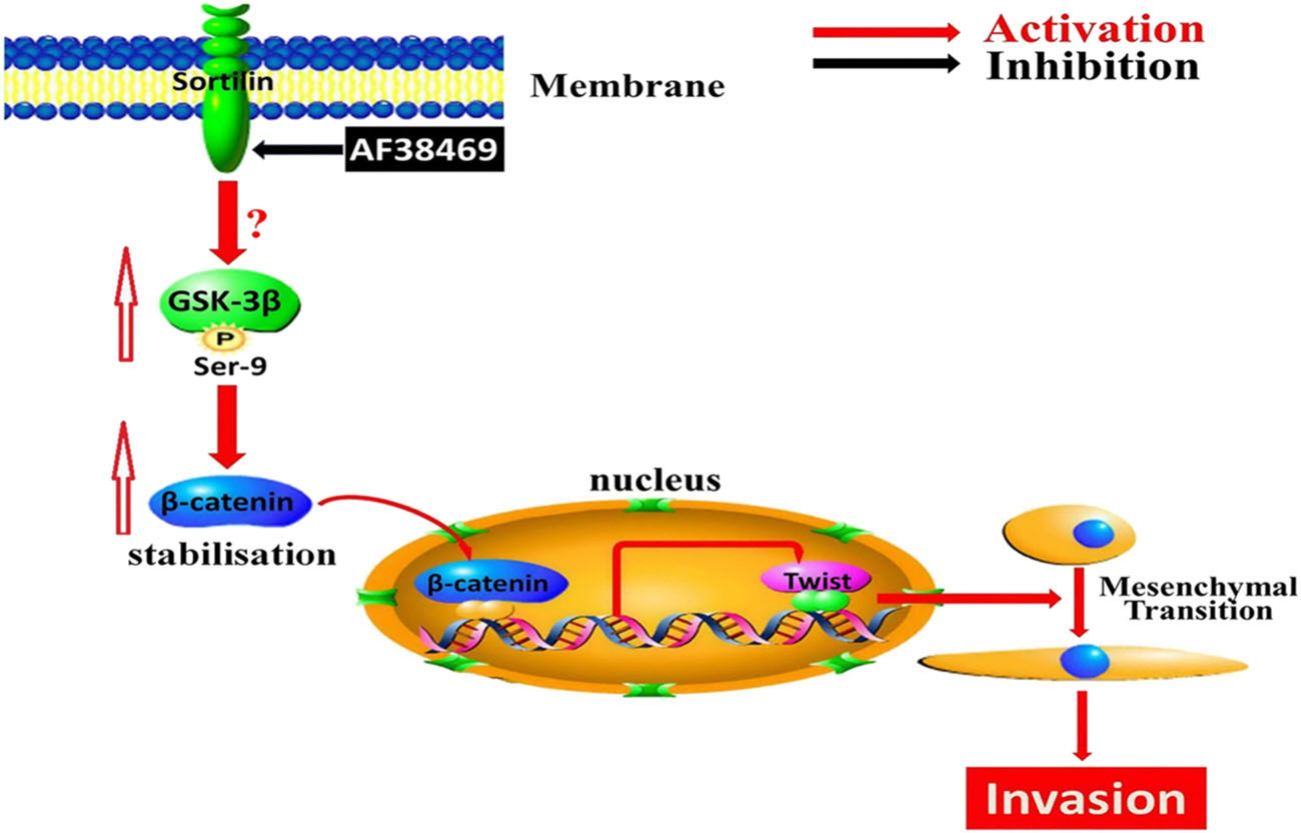
**Schematic model.** Sortilin promotes invasion by GSK-3β/β-catenin/Twist-induced EMT-like mesenchymal transition in human glioblastoma

To further explore the effects of sortilin on GBM invasion and EMT-like mesenchymal transition in vivo, we subcutaneously implanted 1 × 10^6^ U87 cells into nude mice. After 21 days, the tumor volume of subcutaneous GBM in the AF38469 group was much smaller compared with control group (Fig. 7a), indicating that AF38469 attenuates subcutaneous GBM invasion growth. By western blotting analysis, we found that AF38469 decreased the activation of GSK-3β/β-catenin/twist axis in tumor tissues. Meanwhile, AF38469 increased T-cadherin expression, while decreasing the levels of N-cadherin, vimentin, and MMP-9 in subcutaneous tumor tissues (Fig. 7b). Next, we established orthotopic GBM xenograft models by implanting U87 cells into the right striatum and detected the size of the intracranial tumors by MRI scanner. The results indicated that AF38469 significantly decelerated the invasion growth of orthotopic xenografts (Fig. 7c, d). In addition, results from hematoxylin and eosin staining showed that the tumor margin of the AF38469 group was clearer and smoother than that of the DMSO group, suggesting that AF38469 suppresses the invasive capacity of intracranial glioblatoma (Fig. 7e). Kaplan–Meier survival analysis was used to investigate the effect of AF38469 on the median survival time of GBM-bearing mice. We found that the median survival time of mice in the AF38469 group (28.5 days) was significantly longer than that of mice in the DMSO group (18.5 days) (Fig. 7f). These data confirms that sortilin promotes GBM invasion possibly via GSK-3β/β-catenin/Twist-induced mesenchymal transition in vivo, whereas AF38469 impairs GBM invasion and mesenchymal transition, and prolongs the survival time of GBM-bearing mice (Fig. 8).

## Discussion

In this study, our results clearly indicated that sortilin is critical for GBM aggressiveness. We found that the level of sortilin was positively correlated with GBM aggressiveness, and that high levels of sortilin predicted poor prognoses in patients with GBM. Additional results demonstrated that sortilin promoted GBM invasion by enhancing mesenchymal transition in vitro and in vivo. In addition, we confirmed that the GSK-3β/β-catenin/Twist pathway was involved in sortilin-promoted GBM invasion and mesenchymal transition. Our data also suggest that AF38469 might be a promising agent for sortilin-overexpressing human GBM.

High aggressiveness is one of the hallmarks of GBM. The local invasive growth of GBM complicates complete surgical resection and predicts a poor prognosis. GBM invasion is a complex process that involves mesenchymal transition, cell motility, extracellular matrix remolding, etc. Mesenchymal transition is one of the important steps during invasion. In general, human GBM is classified according to histopathological features; however, recent genotyping and expression profiling analyses have demonstrated that GBM can be categorized into four subclasses depending on neural differentiation, including the proneural, neural, classical, and mesenchymal subtypes. The mesenchymal subtype is characterized by high neoangiogenesis and an invasive nature, which predicts poor prognosis in patients with GBM^17^. The importance of this classification is that the different subtypes may require different therapeutic approaches. Mesenchymal transition is a reversible biological process that leads to the acquisition of a mesenchymal phenol type characterized by enhanced cell invasive capacity^18^. In this study, our results confirmed that sortilin levels were high in the mesenchymal subtype of GBM and highly invasive types of GBM tissues and cell lines, and predicted poor prognoses in patients with GBM. Our data suggested that sortilin might participate in GBM aggressiveness. Indeed, we found that sortilin promoted GBM migration and invasion both in vitro and in vivo, but these effects could be attenuated by AF38469. In addition, we observed that both AF38469 and Si-Sor could decrease the levels of mesenchymal-related markers, such as N-cadherin, vimentin, MMP-2, and MMP-9, and increase the level of T-cadherin, suggesting that sortilin could enhance or at least maintain the mesenchymal transition of GBM. Therefore, our data further confirmed the close relationship between mesenchymal transition and GBM invasiveness.

The level of WNT/β-catenin has been shown to be elevated in human GBM and high levels of WNT/β-catenin are related to a significantly shorter survival time in patients. Importantly, evidence has shown that WNT/β-catenin has a vital role in GBM invasion and EMT. In this study, we found that AF38469 or Si-Sor suppressed the levels of p-GSK-3β and β-catenin, whereas the inhibitory effect of AF38469 or Si-Sor on β-catenin expression were reversed by a GSK-3β inhibitor (SB216763) or Si-GSK, suggesting that GSK-3β/β-catenin is positively regulated by sortilin in GBM. Moreover, WNT/β-catenin has been confirmed to be involved in GBM aggressiveness by triggering the expression of EMT activators, such as Twist, ZEB, Snail, and Slug. Indeed, our results demonstrated that AF38469 or Si-Sor decreased Twist expression and this effect could be reversed by SB216763 or Si-GSK, suggesting that sortilin positively regulates Twist expression via GSK-3β/β-catenin in GBM. Further results showed that the inhibitory effect of sortilin knockdown on GBM invasion and mesenchymal transition could be reversed by aGSK-3β inhibitor, indicating that GSK-3β/β-catenin is critical for sortilin-induced GBM invasion and mesenchymal transition. In addition, overexpression of Twist not only promoted GBM mesenchymal transition but also reversed the inhibitory effect of AF38469 on this process, implying that sortilin enhances GBM invasion and mesenchymal transition via Twist. The above data suggested that sortilin enhances GBM invasion mainly via GSK-3β/β-catenin/Twist-induced mesenchymal transition.

The biological effects of NTS are triggered by its interaction with NTSR1, NTSR2, and sortilin. Our previous study demonstrated that NTS and NTSR1 are overexpressed in human glioma, and that the activation of NTS/NTSR1 signaling is associated with the progression of glioma^19,20^. Accumulating evidence indicates that sortilin is highly expressed in many cancers and is involved in the process of cancer development^14,21,22^. Some reports demonstrated a strong staining of sortilin in the cytoplasm and only 5–10% of sortilin on the cell surface^23,24^. Our study further confirmed this observation in GBM. These data show that sortilin might have diverse biological functions in cells. As an NTS receptor, sortilin is involved in the NTS-induced proliferation and migration of human microglial cells^25,26^. In addition, sortilin acts as a co-receptor with RTks and p75^NTR^ to regulate numerous biological processes. Although sortilin is highly expressed in high-grade glioma and is positively correlated with glioma malignancy^15^, the biological function of sortilin in human GBM has not been clarified. In our study, we first demonstrated that sortilin promoted GBM invasion by enhancing mesenchymal transition both in vitro and in vivo. Compared with a series of sortilin inhibitors, such as AF40431, AF38469 has very high solubility and membrane permeability. These features make AF38469 a valuable molecular tool for investigating the biology of sortilin^27^. In this study, we observed that AF38469 efficiently repressed GBM invasion via GSK-3β/β-catenin/Twist-induced mesenchymal transition in vitro and in vivo.

In this study, we focused on the GSK-3β/β-catenin/Twist pathway, because it is widely accepted as the most important signaling pathway in the mesenchymal transition of GBM. However, one limitation of our study is that the mechanism of sortilin-induced GSK-3β phosphorylation in GBM was not investigated in this study. Recently, study implies sortilin is a key regulator of epidermal growth factor receptor (EGFR) internalization and limits the EGFR signaling and loss of sortilin in tumor cells promoted cell proliferation by sustaining EGFR signaling at the cell surface, ultimately accelerating tumor growth^28^. As one of RTK, EGFR strongly correlates with phosphatidyl inositol 3-kinase (PI3K)/AKT/GSK-3β^29^. Thus, sortilin may promote GSK-3β/β-catenin/Twist through repression EGFR/PI3K/AKT signaling in GBM, which will be investigated in our future study. In addition, although AF38469 has strong anti-GBM activity in vitro and in vivo, the ability of AF38469 to cross the blood–brain barrier (BBB) has not been investigated. However, according to previous research, AF38469 has low clearance and high solubility in DMSO, and high membrane permeability, which implies that AF38469 may as well be permeable to BBB^16^. In addition, it is critical for application of AF38469 in intracranial diseases such as human GBM, brain injury, and Alzheimer’s disease^30^. Moreover, we cannot ignore the potential toxicity of sortilin suppression observed in hepatocytes, adipocytes, and neurons^31–33^, and therefore an assessment of the potential risks and benefits of AF38469 is necessary.

## Conclusions

We revealed the oncogenic effects of sortilin in GBM aggressiveness and indicated that sortilin might be a potential prognostic factor for human GBM. We found that sortilin promoted GBM invasion via GSK-3β/β-catenin/Twist-induced mesenchymal transition, and that AF38469 could suppress GBM invasion and mesenchymal transition in vitro and in vivo (Fig. 7). Importantly, we highlighted that AF38469 might be a powerful therapeutic agent for GBM. Although our study has provided insights into the oncogenic role of sortilin in GBM, comprehensive studies describing the upstream signaling molecules and prognostic value of sortilin in GBM patients are warranted.

## Supplementary information

Supplementary figure legends

Figure S1

Figure S2

Cell line certification of A172

Cell line certification of U87
