# Supplementary material for: Sortilin promotes glioblastoma invasion and mesenchymal transition through GSK-3β/β-catenin/twist pathway
Source: Cell Death Dis. 2019 Feb 27;10(3):208. doi: 10.1038/s41419-019-1449-9 (PMC6393543; doi:10.1038/s41419-019-1449-9)
Supplement: Supplementary file 1 — Supplementary figure legends [file 41419_2019_1449_MOESM1_ESM.docx]

Supplementary Fig. S1. **(A)**The levels of sortilin protein in the indicated groups were detected by Western blotting in U87 and A172 cells (Upper), the histogram corresponds to the mean±S.D. of three independent experiment, **p*<0.05.**(B)** U251cell lines were treated with 400nM AF38469 or transfected with Si-Sor. Invaded cells were stained and counted using microscopy, scale bar=200um. **(C)**Western blot to investigate the influence of GSK**-**3β inhibitor SB216763 on the expression of sortilin in U87 cells. **(D)** Western blot to investigate the effect of Twist over-expression on GSK-3β, β-catenin and sortilin in U87 cells.

Supplementary Fig. S2. **(A)** Representative image of transwell assays when Si-GSK-3β transfected or combined with AF38469 treatment in U87 and A172 cells, scale bar=200um. **(B)** Representative results of transwell assays when Si-Twist transfected or combined with AF38469 treatment in U87 and A172 cells, scale bar=200um.**(C)** Transwell assays to detect the effect of SB216763 or combined with AF38469 on invasion ability of U251 cell, scale bar=200um.**(D)** Transwell assays to detect the effect of lv-Twist transfected or combined with AF38469 treatment on invasion ability of U251 cell, scale bar=200um.
