# Supplementary figures and images for: Sortilin promotes glioblastoma invasion and mesenchymal transition through GSK-3β/β-catenin/twist pathway
Source: Cell Death Dis. 2019 Feb 27;10(3):208. doi: 10.1038/s41419-019-1449-9 (PMC6393543; doi:10.1038/s41419-019-1449-9)

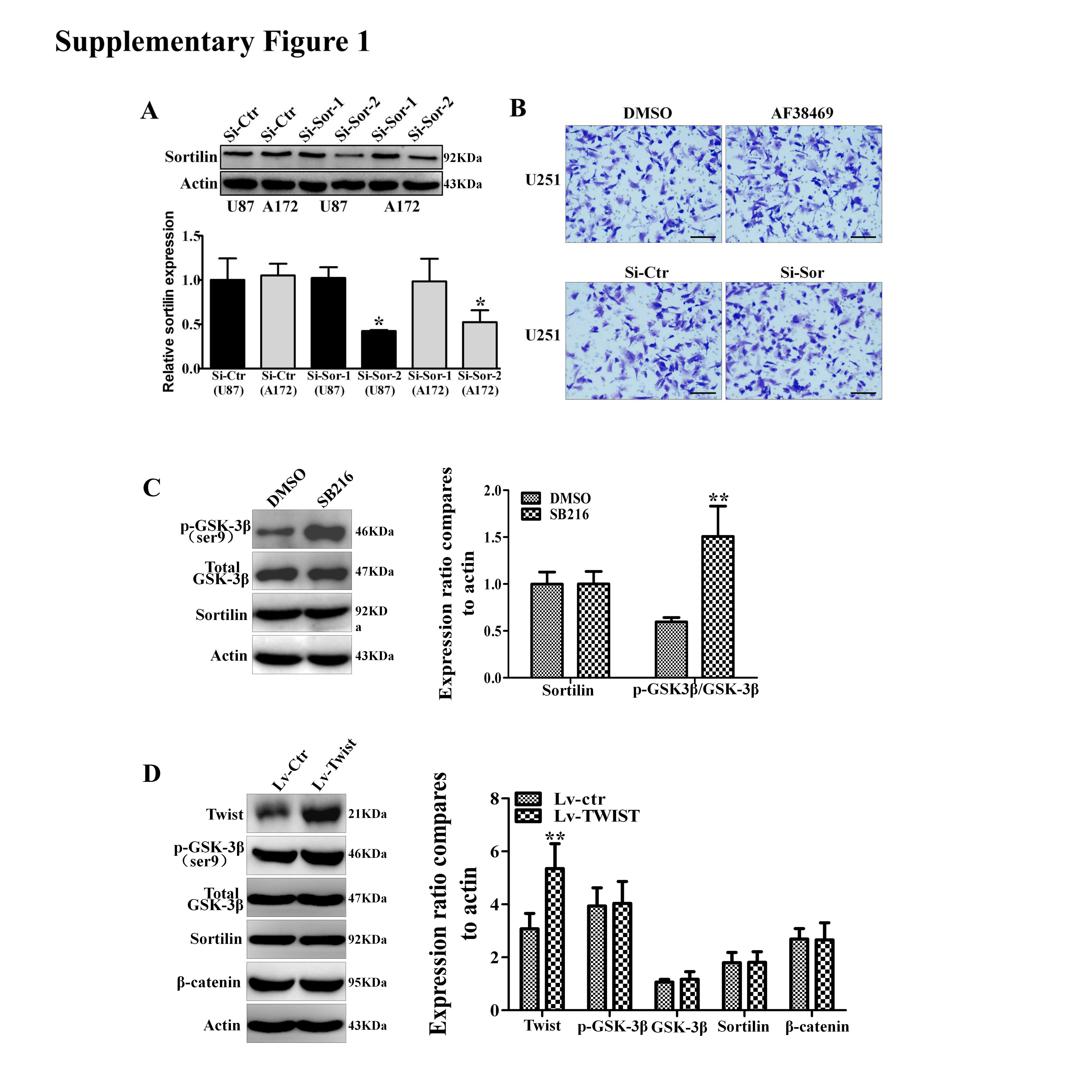

Supplement: Supplementary file 2 — Figure S1 [file 41419_2019_1449_MOESM2_ESM.jpg]

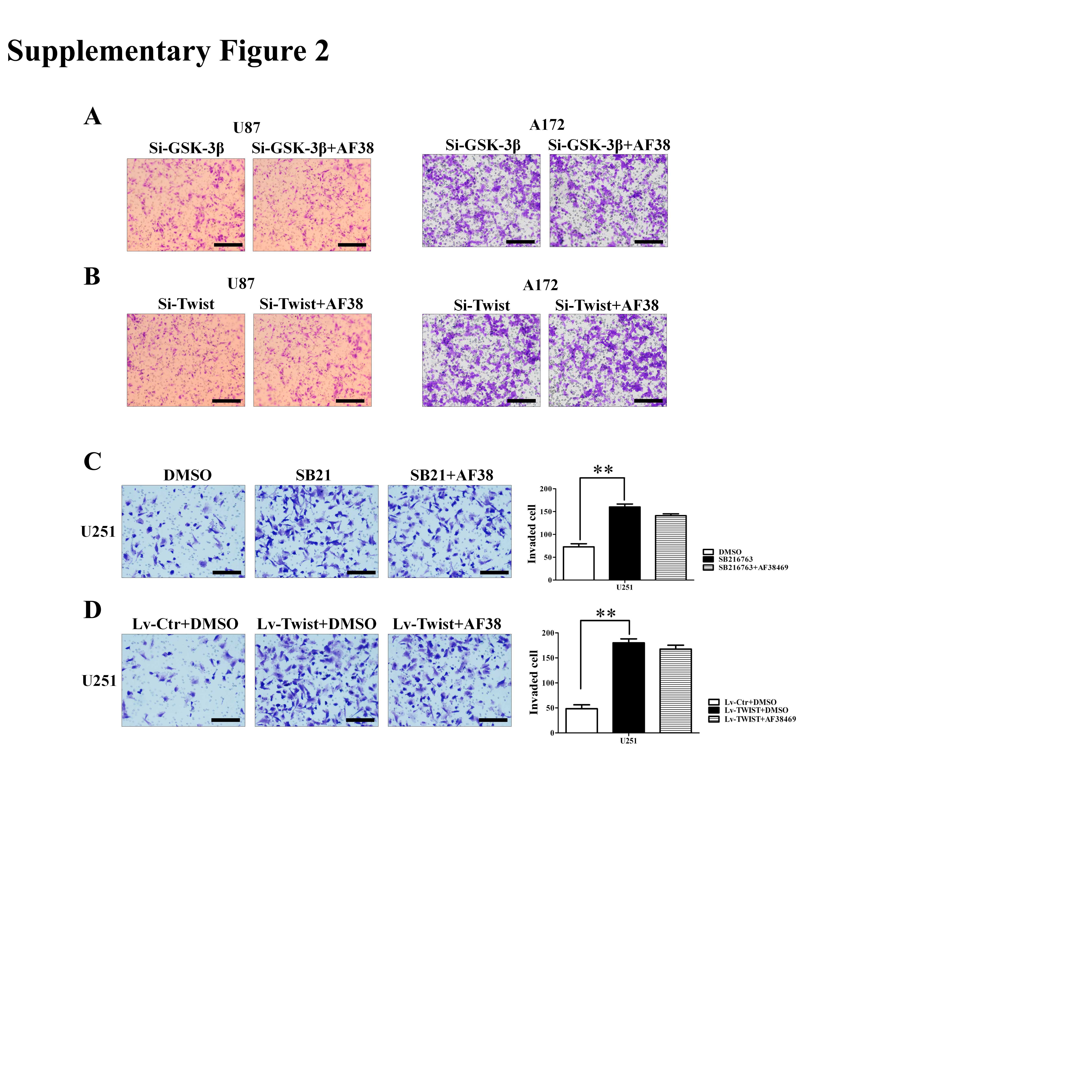

Supplement: Supplementary file 3 — Figure S2 [file 41419_2019_1449_MOESM3_ESM.jpg]

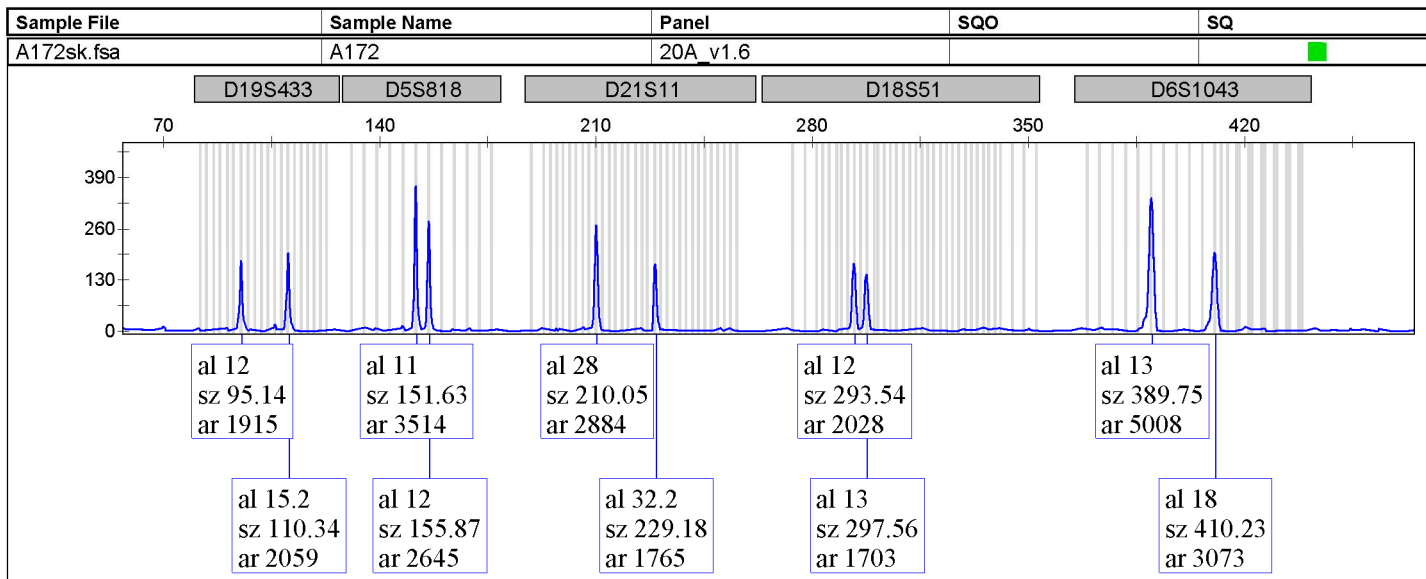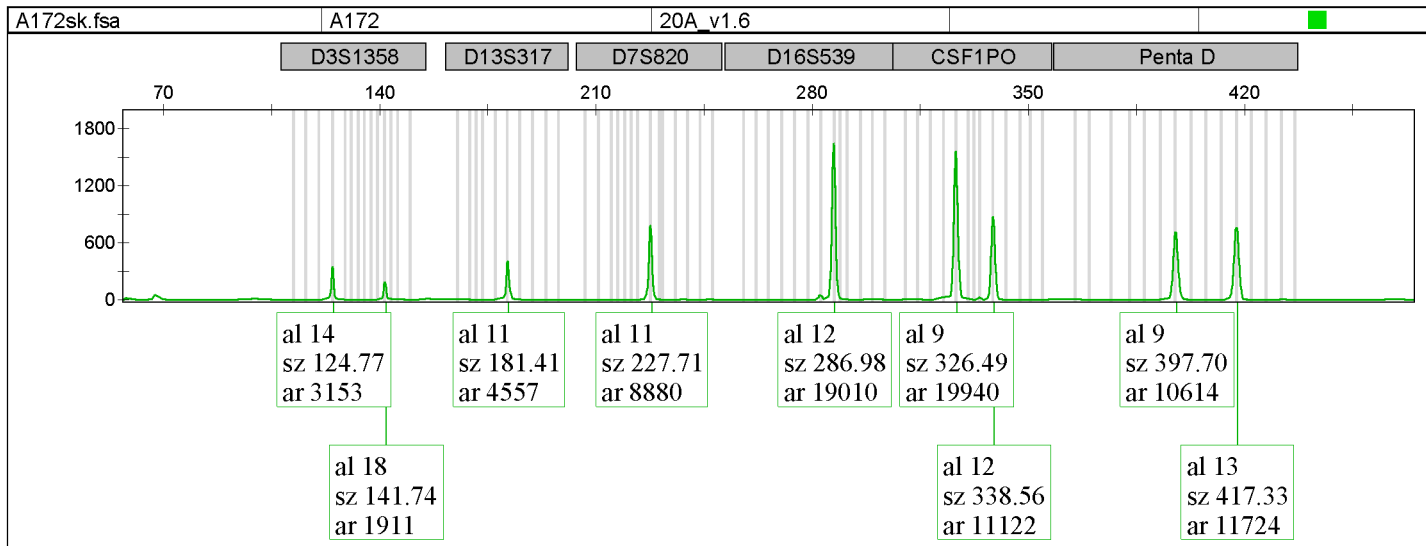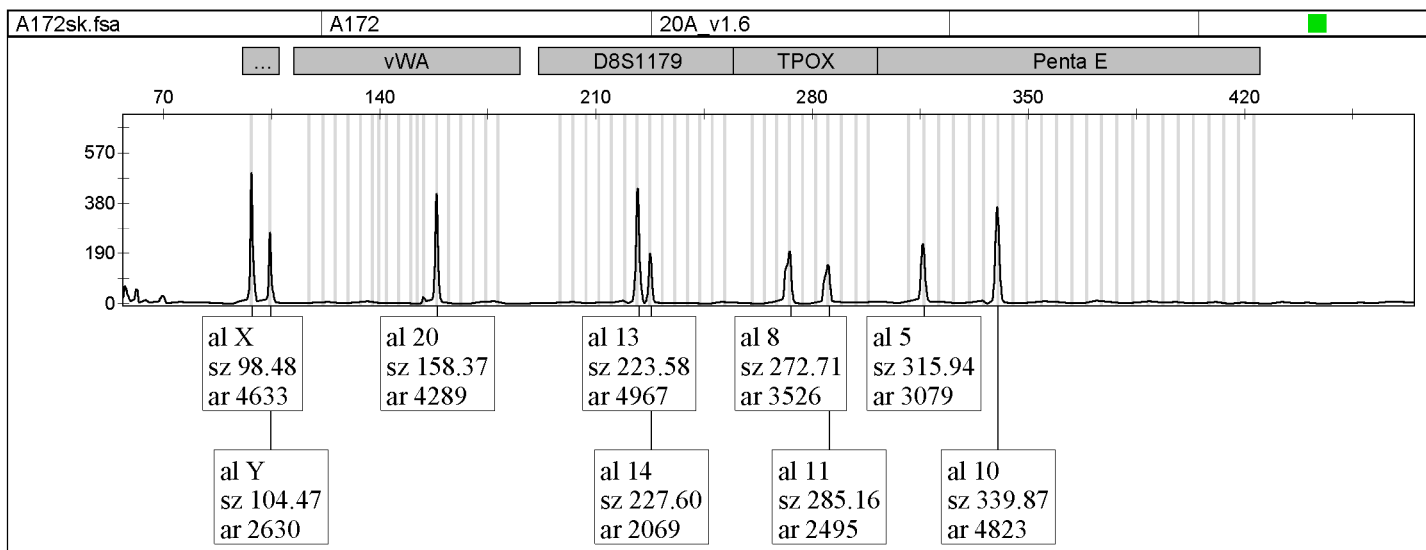

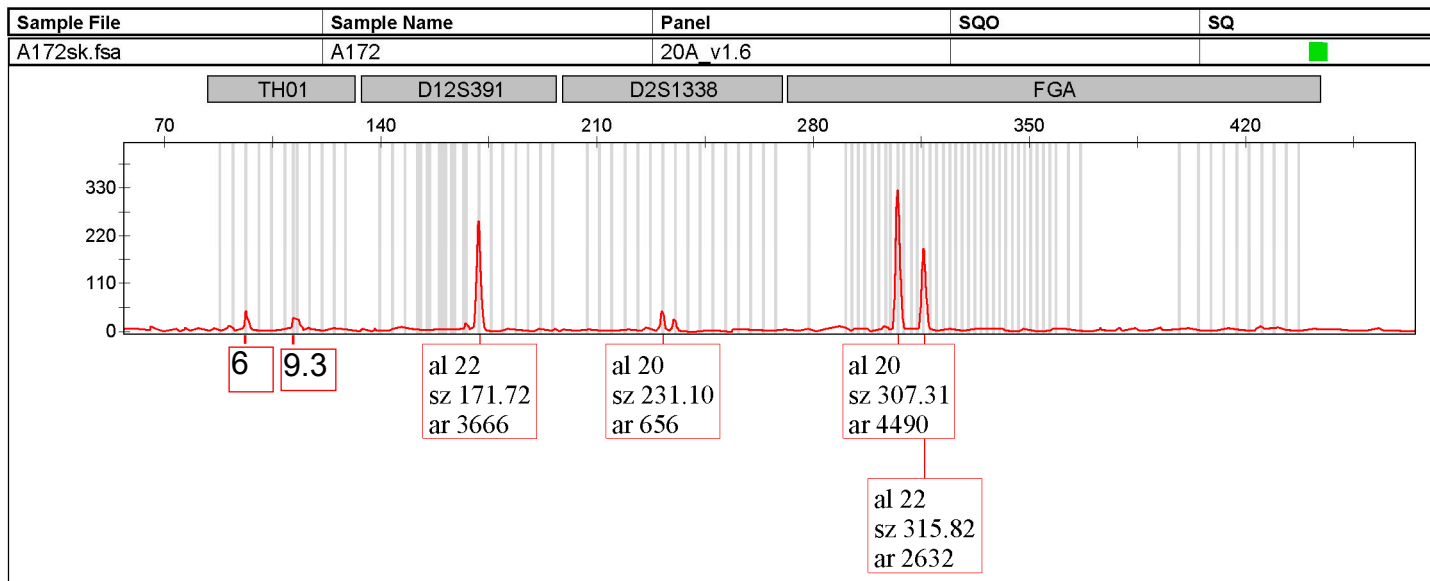

Supplement: Supplementary file 4 — Cell line certification of A172 [file 41419_2019_1449_MOESM4_ESM.pdf]

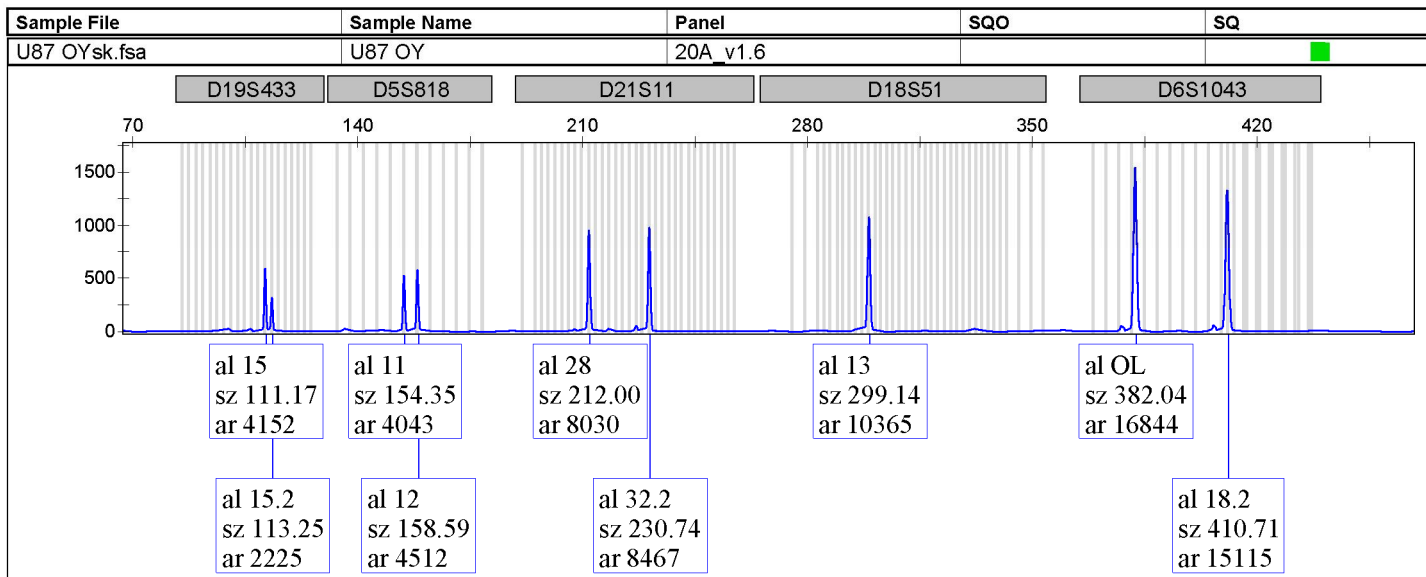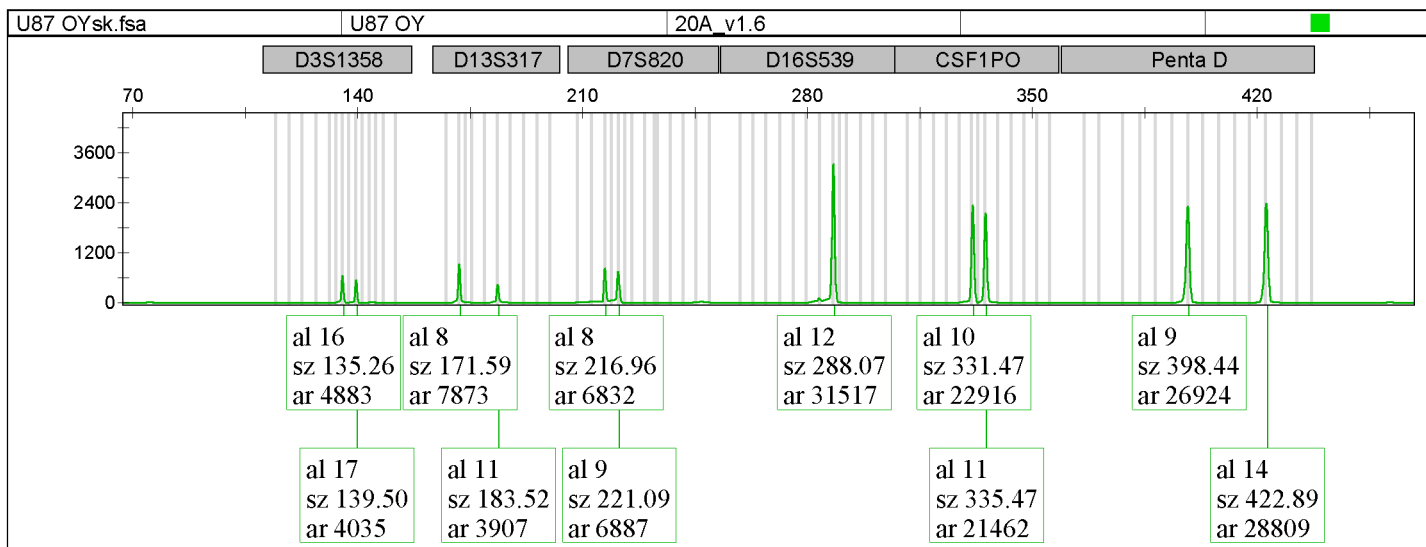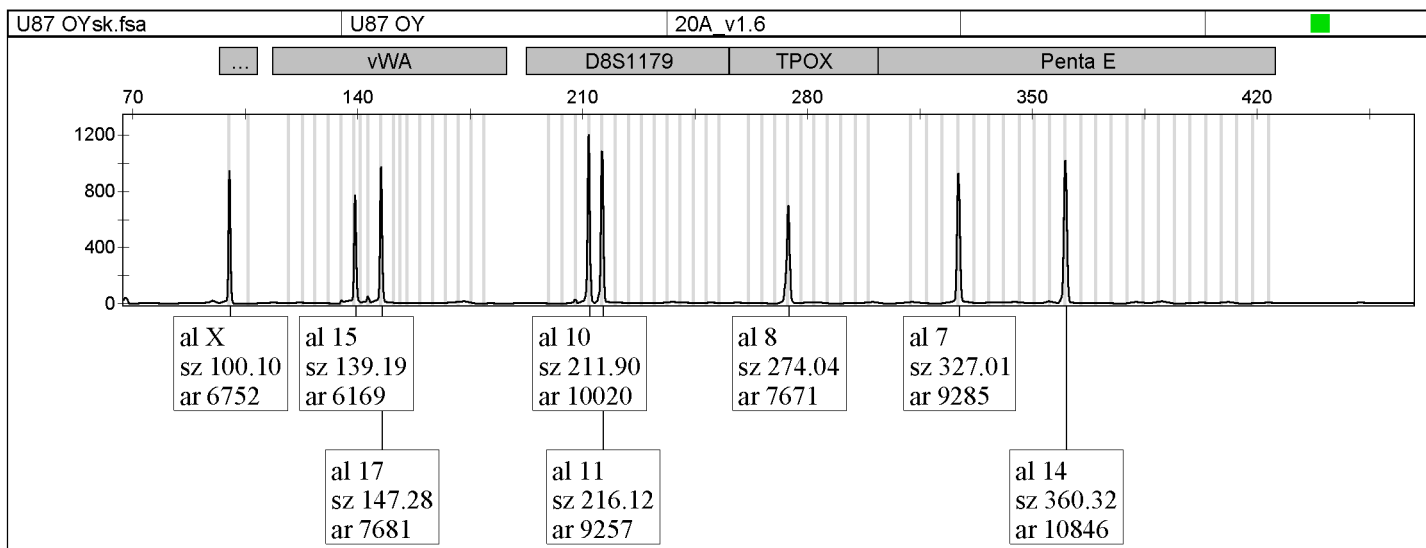

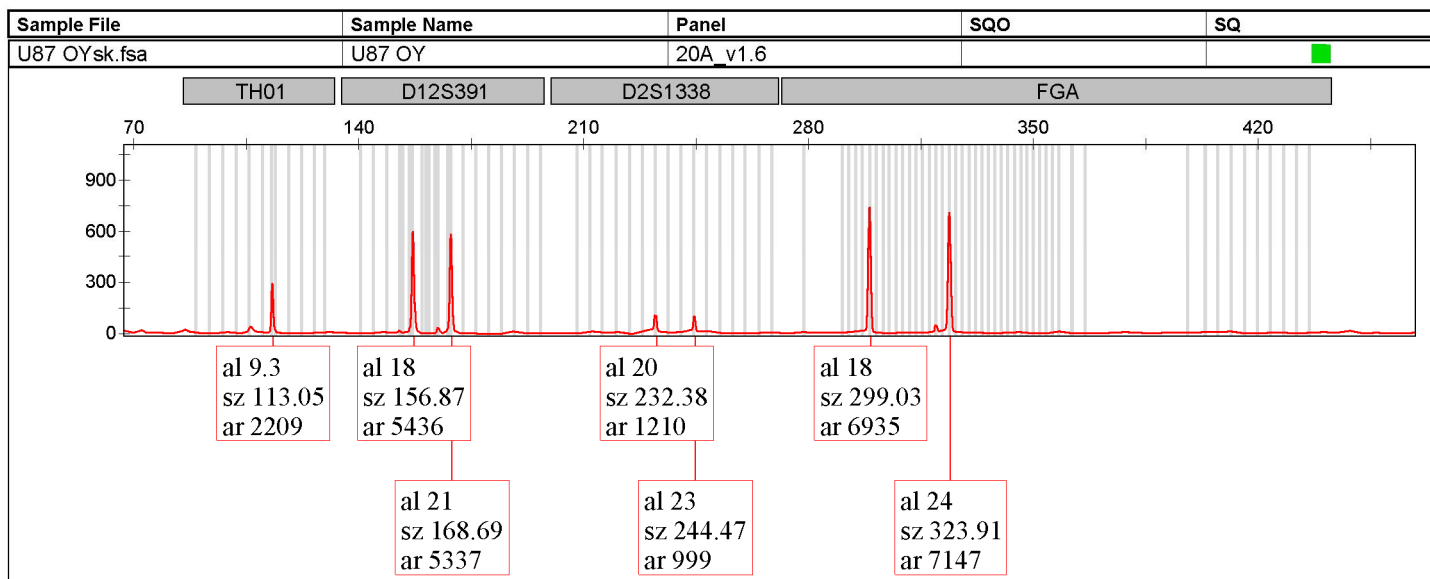

Supplement: Supplementary file 5 — Cell line certification of U87 [file 41419_2019_1449_MOESM5_ESM.pdf]
